# Normative uncertainty and societal preferences: the problem with evaluative standards

**DOI:** 10.3389/fnrgo.2023.1147211

**Published:** 2023-07-17

**Authors:** Sietze Kai Kuilman, Koji Andriamahery, Catholijn M. Jonker, Luciano Cavalcante Siebert

**Affiliations:** ^1^Intelligent Systems Department, Faculty of Electrical Engineering, Mathematics & Computer Science, Delft University of Technology, Delft, Netherlands; ^2^Department of Industrial Engineering and Mechatronic Systems, IMT Mines Alès, Alès, France

**Keywords:** normative uncertainty, limit of forms, ethics, preference profiles, moral machine, self-driving cars

## Abstract

Many technological systems these days interact with their environment with increasingly little human intervention. This situation comes with higher stakes and consequences that society needs to manage. No longer are we dealing with 404 pages: AI systems today may cause serious harm. To address this, we wish to exert a kind of control over these systems, so that they can adhere to our moral beliefs. However, given the plurality of values in our societies, which “oughts” ought these machines to adhere to? In this article, we examine Borda voting as a way to maximize expected choice-worthiness among individuals through different possible “implementations” of ethical principles. We use data from the Moral Machine experiment to illustrate the effectiveness of such a voting system. Although it appears to be effective on average, the maximization of expected choice-worthiness is heavily dependent on the formulation of principles. While Borda voting may be a good way of ensuring outcomes that are preferable to many, the larger problems in maximizing expected choice-worthiness, such as the capacity to formulate credences well, remain notoriously difficult; hence, we argue that such mechanisms should be implemented with caution and that other problems ought to be solved first.

## 1. Introduction

One of the many consequences of technological development that we can observe is the increasing level of deployment of AI systems. The encroachment of such systems is becoming more pervasive throughout all areas of life. However, the differences the variety of environments in which AI systems are deployed should be noted. Recommendation systems for document retrieval may have a limited range of possible outcomes; in contrast, technology like that used in self-driving cars and automated weapon systems needs to act on a far more open-ended environment.

An increase in deployment in these kinds of environments may come with the introduction of disastrous errors. Not only are these environments often associated with higher stakes, they are also far more difficult to observe correctly and completely. We can consider the issue of missed inferences, e.g., missing important and relevant details in a given situation. Agents may need to act under a kind of empirical uncertainty (not having all the information) but also under normative uncertainty (not knowing what one ought to do). For example, when considering self-driving cars, we may need to adhere to incomparable human values. Do we prefer the values of the passengers or those of bystanders? If we prefer the former, should they be able to halt the car at any point in time, even if it is in the middle of high-speed traffic?

These problems create a need for individuals and society to have some form of acceptable or meaningful control over such systems, such that they can be implemented effectively, and so that responsibility gaps can be avoided (Matthias, 2004). Furthermore, the value of such control may also lie in ensuring that these systems are better aligned to individual and societal values.

The problem with control in terms of normative uncertainty, however, is that we need to understand whose choices should be paramount and why. Aside from the fact that any autonomous system may lack access to relevant data, we need to understand that a kind of value pluralism needs to be addressed as well. The main question is: how should an AI agent infer which “ought” it needs to maintain? When given a choice between option A and option B, it needs to have a certain amount of knowledge of the world and to know whether A or B should be preferred. Therefore, this question does not merely revolve around the data, but also requires us to be able to resolve conflicts between sometimes incommensurable beliefs.

Of course, if we ask the broad question of how *we* ought to act, then we can draw from a deep well of philosophy and normative ethics regarding what a correct action entails. We can think of virtue ethics, whereby we need to be a virtuous agent, or deontology, under which our intentions reign supreme; or we can think of consequentialism, where the consequences of our actions matter most. Even further from such obvious theories of correct action, the answer to this broad question can also be found in Seneca, Marcus Aurelius, Nietzsche, and many other works. Of course, in these cases, the question of how we ought to act is then taken to be relatively similar to the question of how to be in the world.

The questions and debate surrounding normative uncertainty^1^ are still relatively new (Lockhart, 2000; Sepielli, 2010). They are based around the assumption that we ourselves may not know the correct course of action and must thus navigate in the dark (Sepielli, 2010). In economics, people have made the comparison to the “original position” as proposed by Rawls: in such a situation, we need to decide how to share out the economical cake, yet we do not know what position we might hold in society. Our identity in the original position is uncertain, and yet we must make decisions—often with socioeconomic consequences (Dietrich and Jabarian, 2018).

Theories surrounding normative uncertainty have provided some ideas as to how to go about dealing with it. Mostly these involve maximizing expected choice-worthiness (Sepielli, 2010; MacAskill and Ord, 2020): in this case, we should conceptualize possible belief systems as different worlds and see what their outcome is. For example, we can consider two worlds, one in which it is morally wrong to harm an animal and one where it is not. If we need to choose between eating meat and not eating meat, then we can argue that, given these possible worlds, not eating meat is going to prevent the harm either way (it is the most choice-worthy option).

However, for AI systems, we are not dealing with personal belief systems but rather with a plurality of values, societal norms, and differing interpretations. How do we control such a system, and what “oughts” should it adhere to—and whose “oughts,” in cases of such plurality? In this article, we demonstrate the possibility of applying Borda voting to normative uncertainty as one approach to maximizing expected choice-worthiness among a crowd. Normative uncertainty has been previously related to a voting problem (MacAskill, 2016), where Borda voting was proposed as a solution to the different possible moral theories that individuals could adhere to. However, thus far, this proposition has lacked empirical analysis of what that would imply when applied to people's preferences. We provide such an analysis by virtue of a very simple experiment. Our objective is to show how one can counteract normative uncertainty within agents through the use of preference elicitation from individuals. For our domain, we use data from the Moral Machine experiment (Awad et al., 2018), but this is merely intended as a working example, as these data provide people's preferences in an AI-infused domain and are widely available. While the Moral Machine experiment was criticized for its inauthenticity or its lack of relevance to morality, we argue that the experimental results are not to be taken at face value but rather have broader applications, as they may provide an opening for discussion of credences and value-sensitive design processes. We also add to the discussion by introducing what we deem to be the most important and pressing issues for such a solution.

In this article, in summary, we review the ethical theories that we formalize, explain how we formalize them, and then present results on the differences between various voting systems. Subsequently, we analyze the results and discuss some of the larger issues involved in attempting to model these intricate beliefs.

## 2. Method: reading the road signs

MacAskill describes the problem of maximizing expected choice-worthiness under normative uncertainty as a voting problem, for which he suggests that Borda voting (a modified form of ranked-choice voting) may be preferable as a solution (MacAskill, 2016). The premise of Borda voting, as defended by MacAskill, is that it is effective in avoiding choices that are considered unacceptable. It is a type of voting in which the success of an option is calculated as the sum of its pairwise victories against all other options. For a single individual aiming to make a decision based on ethical principles, we take this to mean that they should seek out all possible ethical theories, giving credence to the possibility of a variety of options, and then assess which option is the most acceptable most of the time. For AI systems, these rankings are not formulated by an individual but rather by a host of data sourced from different individuals; this means that which option is acceptable most of the time is dependent not on the ethical theories themselves but rather on differences in credences between individuals.

To illustrate the effectiveness of Borda voting for agents with multiple stakeholders, we base our argument on an experiment built upon data from the Moral Machine experiment conducted in 2018 by MIT (Awad et al., 2018) as a source of data regarding human preferences. In the Moral Machine experiment, the moral choices of respondents across the world were collected through an online survey. They were required to make decisions in response to variations on the following scenario: an autonomous vehicle begins to have trouble with its brakes just before arriving at a crosswalk. Because of this failure, the autonomous vehicle cannot avoid an accident and must choose between two lanes by either staying on course or swerving. This is well known as the trolley problem. In this study, we repurpose the data from the Moral Machine experiment, based on the well-known trolley dilemma, to explicate the credences that individuals may have toward selected ethical theories.

### 2.1. The moral machine dataset

The Moral Machine experiment collected data from 1.3 million respondents from 233 countries and territories. Although 13 scenarios were presented to each respondent, some of them did not answer them all. In our study, we consider the subset of respondents who provided answers to all 13 dilemmas, corresponding to 51,122 users. Respondents who answered multiple times were also disregarded.

The aim of our study was to measure individuals' preferences from an ethical perspective, by drawing up a moral profile for each respondent. As each of them faced 13 different variants of a modern trolley dilemma, we measured their preferences for each of our operationalized theories by determining how many times a principle was either followed or disregarded in their decisions. We therefore analyzed the relevant attributes of each dilemma presented in the experiment. These were: the characters involved (pets or humans); their age (young, adult, or senior); their role (pedestrians or passengers of the autonomous vehicle); the number of characters on each side of the road; whether or not any pedestrians were law-abiding; and two additional specific attributes (the presence of pregnant women and strollers). There were a few other attributes presented in the experiment that we did not consider: social status, gender, fitness, and specific education (e.g., doctors). The reason for disregarding these was that the theories involved do not differentiate based on these attributes.

### 2.2. Selected ethical theories

To effectively distinguish between different ethical possibilities, we need to draw some distinction between ethical theories. For this study, we mostly drew on different versions of utilitarianism and some deontological principles as inspiration for the proposal of a set of operationalizable ethical principles. Below, we present what these principles should represent; later, in the implementation section, we return to the question of what they actually entail in terms of practical choices.

**Sentient utilitarianism**: This principle favors choices that minimize the number of lives that are lost, giving equal consideration to all sentient beings and not just to humans in particular.

**Classical utilitarianism**: This principle promotes choices that minimize only the number of human lives that are lost, and thus other types of beings are not considered.

**Hedonistic utilitarianism**: This principle encourages choices that maximize years of human life in order to promote more human pleasure in the long run. The corresponding theory considers hedonism, which is the idea that pleasure is the only good thing in itself, and measures pleasure in terms of a specific unit called the “hedon.”

**Agent-centered deontology**: The agent in our study is the autonomous vehicle. This principle dictates that there is some obligation for an agent to take certain actions and/or to refrain from taking others. Since killing is a morally reprehensible action, the agent should do anything in order to avoid actions that lead to the deaths of others.

**Patient-centered deontology**: Under this principle, rights are promoted to a greater extent than duty. The patients in this case are considered to be every agent (animals and humans) apart from the autonomous car—and, inevitably, people inside it. The right we consider here is the right not to be killed intentionally. Because the trolley dilemma ineluctably deals with the death of at least one being, the autonomous car should not swerve under this principle, because that would be seen as taking the action of killing people on the other side of the road.

**Contractarian deontology**: This principle is based on considering laws as moral rules for taking particular actions, and therefore penalizes illegal behavior.

In order to choose which moral principles to focus on, we made some simplifications that are justified in the following section. Some assumptions were also made because not all the criteria in the dataset were relevant or sufficient for a moral principle to fit perfectly. We should also note the glaring lack of virtue ethics, which is a system that is difficult to operationalize well. The possible consequences of this are discussed in Section 4.

### 2.3. Voting systems

Our goal was to apply voting methods to enable an artificial agent to deal with normative uncertainty due to the preferences of multiple stakeholders. Given our proposed set of ethical theories, we can estimate the extent to which each stakeholder believes in each of these theories, if not precisely, then at least sufficiently to rank them in order.

The Moral Parliament, as introduced by Bostrom (2009) and further developed by Newberry and Ord (2021), considers the construction of a moral parliament. Under normative uncertainty, this parliament invites a variety of delegates from different moral theories to represent the ethical principles of their corresponding framework. The intention is that the parliament should engage in debate in order to determine which choice to make. In this case, rather than a debate, we take the data already provided by participants and show how they cast their ballots by making a choice in the Moral Machine experiment. A decision in the parliament can be made through different voting schemes. In our study, we consider three such schemes.

**The majority vote**: In a classic majority count, alternatives are voted on by each group of delegates. In each instance, the one that receives more votes wins.

**Borda count**: As defended by MacAskill (2016), the Borda method may appear to be efficient, as it avoids choices that are considered unacceptable. The Borda count employs a ranked voting system, in which points are allocated to each alternative.

**“My favorite theory”**: As presented by Gustafsson and Torpman (2014), my favorite theory (MFT) can be stated as follows: an option *x* is only permitted in a situation *S* for an individual *P* if said individual *P* has the highest credence in a particular theory and that theory permits option *x* in situation *S*. In voting, this means that the largest group of delegates always gets to decide the outcome. This theory prescribes the most consistent choices without overly relying on inter-theoretic comparisons.

Using the data from the Moral Machine experiment on the choices that individuals made during the experiment, we can construct a hypothetical parliament. This hypothetical parliament can convene and show what outcome they would arrive at, depending on the voting scheme employed. Furthermore, this can tell us something about whether principles are violated or respected.

### 2.4. Implementation

To fully comprehend the possibilities of a moral parliament and Borda voting as a solution for moral uncertainty, we needed to formalize the preference profiles. In order to operationalize our chosen ethical theories, we focused on the most relevant attributes, as some of them do not affect behavior under all ethical theories. In addition to that, we needed to make certain simplifications and assumptions in order to correctly evaluate which decision would be best to take according to a specific ethical principle. Moreover, it appears that certain scenarios presented to the respondents were designed to test responses to a specific attribute by varying the chosen factor while holding others constant. Therefore, for each defined principle, we incorporated a specific metric we refer to as “ambiguity,” which was used to designate situations where we could not determine whether the moral principle was being followed or not. Ambiguity appears in a proportion of dilemmas where the moral principle in question does not clearly prescribe or forbid the user's decision. Incorporating the situations in which ambiguity appears, we measure what we call a moral profile, which indicates the extent to which a respondent follows each ethical theory. The moral profile of an individual consists of their moral score for each of our ethical theories. The moral score for each theory is defined as the percentage of choices made by the respondent that clearly fit what it promotes.

We formalized each of the preference profiles as follows:

**Sentient utilitarianism**: *max*(*lives*), no matter what kind.

**Classical utilitarianism**: *max*(*human* *lives*).

**Hedonistic utilitarianism**: *max*(*years* *lived*). The data provide no specific details on the number of years of life saved or lost, only the categories of children, adults, and seniors. Therefore, we assumed children to be 10 years old, adults to be 35, and seniors to be 60, and considered the life expectancy of each individual to be 85.

**Agent-centered deontology**: Choose self-sacrifice over involving pedestrians.

**Patient-centered deontology**: Choose self-sacrifice over involving pedestrians; however, when this is not possible, avoid swerving the car.

**Contractarian deontology**: Save pedestrians who are legally crossing the street.

For each situation, a given choice could violate a principle, respect it, or be ambiguous with respect to it. If the principle is violated, the choice goes directly against the preferred outcome; if it is respected, the agent acts accordingly. In some cases, where neither of these was the case, we deemed the choice to be ambiguous: e.g., if both options targeted only people walking through a red light, then from the perspective of contractarian deontology (which cares about law-abiding behavior), it would not matter which choice was made. The principle would be neither violated nor respected in either case.

To give this a little more substance, consider a scenario in which there is one pedestrian on one side of the road and an obstacle on the other, and there is also one person in the car. Under sentient utilitarianism, this is an ambiguous case, as both options cause one party to die. According to agent-centered deontology, however, the car ought not to hit the pedestrian but the barrier instead.

To form a parliament, we need an adequate number of hypothetical individuals who get to “vote” on how the car ought to behave. Using the Moral Machine data, we calculated how many choices followed one principle or another (a choice could follow multiple principles). Based on these results, we calculated the credences of each individual toward their principles. We averaged these principles over the entire population and then, proportional to their credences within the population, each principle was awarded a number of delegates who were assigned to the moral parliament. This parliament always consisted of 100 individuals.

For Borda voting, the members of parliament were required to allocate some number of points to each alternative (two alternatives in this case) in a given situation. Each member was permitted to award 1, 0, or −1 as a score to each alternative depending on whether they found the alternative preferable.

If we compare Borda voting to majority voting, then in this case, the results for a single choice between A and B would likely be relatively similar. However, if we consider the principles as the driving force, then we can understand that ambiguity starts to play a major role. In Borda voting, ambiguity is given a specific role, whereby members of the parliament could award these options 0 points. Overall, due to this subtle change in voting, we expected that more principles would be respected and fewer violations would occur under this scheme^2^.

## 3. Results of the experiment

**Figure 1:**
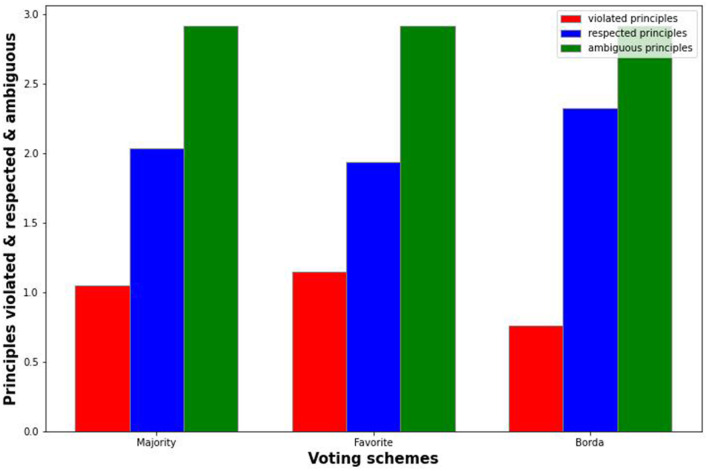
Results of voting based on individual credences.

**Figure 2:**
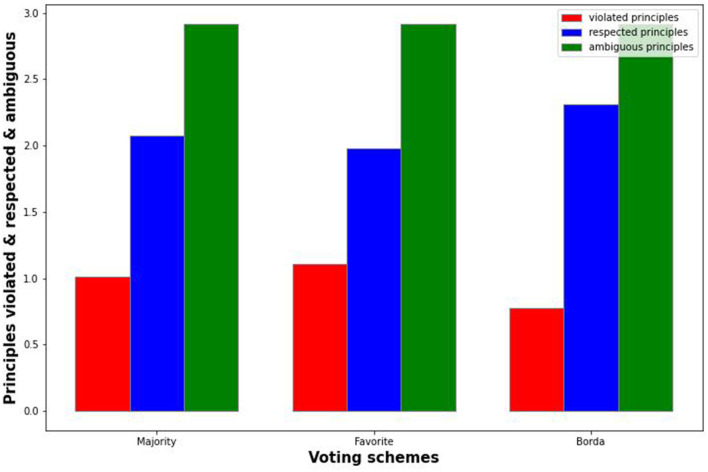
Results of voting based on average credences.

In this section, we present some of the results of the experiment. We make a distinction between individual credences and average credences. For the case of individual credences (see Figure 1), we formed a parliament based solely on individuals' credences^3^. For the case of average credences (see Figure 2), we looked at a societal scale averaging all individual preferences for certain theories, and formed a parliament based on these credences.

The graphs illustrate how many principles the proposed parliament would, on average, respect, and violate. This was based on the entire set of responses. Since there were six possible theories whose principles could be respected or violated, there were also numerous cases in which a principle was neither respected nor violated. The total for these schemes thus always summed to six.

The standard deviation was quite large in all cases (for individuals and also for society^4^); this is to be expected given the wildly differing situations that could occur. Given the 50,000 responses, there was a wide variety of situations, and considering the set of ethical theories from which the proposed parliament might select a theory, there were plenty of opportunities to violate or respect certain principles. This did not represent a flaw in the code or in the data, but is merely an illustration of the wide variety of scenarios that could occur. The standard error, however, was negligible, so we have omitted it.

Although we could delve into the significance of Borda vs. MFT or majority voting, the large standard deviation poses some questions. Perhaps we should have formalized the ethical theories in a different way and considered other attributes of the scenarios, such as the characters' gender or social status. However, because these are socially sensitive, and prescriptive ethical theories tend to concern themselves with other factors, we left them out^5^. We also do not pretend to have exhausted the possible space of theories, nor do we suggest that no other compositions of theories are possible. There are numerous “sub-theories” that could additionally have been examined; the main point of contention is that these systems will necessarily show edge cases that may be undesirable, no matter the type of voting system used. We could discuss the significance of the results and whether Borda is significantly better than MFT or majority voting; however, we propose that this would be misleading (even if we were to formulate this significantly more accurately in terms of respecting principles). In the discussion, we go into further detail with regard to the limits of this.

## 4. Discussion: speeding ahead?

From our current vantage point, it may be simple to posit that Borda voting is preferable to majority voting because it captures more of the nuance of the distinction between choosing and abstaining. Given a moral parliament and the premise of maximizing expected choice-worthiness, it seems to be a good way forward, especially considering the fact that it also specifically avoids the worlds we should disprefer.

Nonetheless, as we mentioned in the Results section, there are some broader issues of control that arise due to the concepts involved. These do not merely stem from Borda voting, but also have to do with some of the premises of maximizing expected choice-worthiness and a technical approach to achieving it.

### 4.1. The limits of form: maximizing garbage

One real problem with normative uncertainty and maximizing expected choice-worthiness itself lies in its formulation. This is known as the limits of form problem (Sepielli, 2010)^6^. When we, as developers, model the world for an agent, we necessarily make abstractions and thus leave out (relevant) details. In maximizing expected choice-worthiness, we may give credence to certain possible worlds (e.g., ones in which animal welfare matters). However, what we take into account and what we do not is roughly determined by our outlook on life and by the extent of our knowledge. In this regard, as a prescriptive moral framework it lacks substance and is difficult to differentiate from My Favorite Theory. If put rubbish in, we get rubbish out. We may be capable of conjuring up possible worlds in which certain theories are true, but which theories we consider in this process is up to us. To hearken back to the previous sections, we included ambiguity as an option for Borda voting because the response always involved a choice between A and B, but that simple choice is also a direct limitation imposed by the dataset: responses were a choice between A and B, while in reality that simply may not be the case. In the data, respondents made a difficult choice between two possible worlds, in which they were required to choose to hit one party or another; but that choice in and of itself may be completely unrealistic. It is far more likely that this should also include the probability of individuals dying, or partial deaths of a group. Instead, we are left with “garbage in” on the level of the data, but also on the level of principles—given the glaring absence of virtue ethics, which we refrained from formalizing because it does not formalize well. It should be no surprise that the maximization of choice-worthiness is limited by all of these factors. The limits of forms therefore make it very difficult to claim that any of these voting systems is a significant improvement over the others, as this would mean that they could be compared in a meaningful fashion. Since these are choices about who dies in which situation, based most likely on insufficient and incorrectly scoped data, it becomes difficult to defend the idea that Borda in fact respects more principles on average, because the relevant question is not only whether it respects more principles to a significant extent, but also whether it behaves in an acceptable manner given an edge case. Overall, the limits of form show that these voting systems and the principles included in them require additional grounding in order to function as effective prescriptive models.

### 4.2. Formal uncertainty

While maximizing expected choice-worthiness may be acceptable and rational for individuals to stumble along with, we need to understand that we introduce a second type of uncertainty with artificial agents. On the one hand, we have already mentioned that only operationalized versions of ethics matter, which means that certain people are unrepresented in the proposed parliament (or at least not represented to the same extent)^7^. On the other hand, we are also opening ourselves up to questions of whether or not the agent is misinterpreting the action itself.

The first issue is simple to explain. Formalizability may be a problem for those whose voice is insufficiently represented by such factors. We can think of holistic attitudes, for example, which are by their very nature in conflict with the featurization of certain systems. People with such attitudes may not be given a seat at the table, which can cause trouble for certain groups. We also know that certain minorities may be overrepresented in the data, and may thus skew the outcome unjustly. MacAskill mentions that a potential solution on a practical level might be search spaces, as these may distinguish the possible alternatives and the “regions” they inhabit. Non-overlapping alternatives are then supposedly considered, such that most of the space can be covered. Our main concern here is that the alternatives themselves as described may still influence the results, as any composition of alternatives will neatly ignore a set of individuals who are not represented by them (however this is carved up). This would be akin to saying that the map is more important than the territory. One can disagree, of course, but to fully explain this would take us far beyond the scope of this article^8^.

The second type of uncertainty is more subtle. In short, we do not always know how an action taken by a system has come about. This can be seen with inappropriate induction of functions (such as classification of wolves as “snow in the background,” Ribeiro et al., 2016). Unless we have access to the chain of decisions and how it came into being, we may be presented with the wrong conclusion, or with the right conclusion for the wrong reasons. For example, it could be that a system might learn to classify something like “being halfway across the crosswalk” as “legally crossing the road” if the data lacked counterexamples. If contractarian deontology then devolves into a strategy of saving the greatest number of pedestrians who are halfway across the crosswalk, then our principles mean nothing.

This means that, in the case of agents, maximizing choice-worthiness may also entail ensuring that its *actions* are actually aligned with the most choice-worthy option. While the issue of the limits of form poses a problem for prescription, formal uncertainty asks whether the prescriptive ideals are executed correctly, as the question is not only how one ought to act, but also how one will act. And while one may argue that we are the ones who provide the input in relation to the credences—these are our suggestions, not the agent's—it is still the agent's execution, and thereby the effective interpretation of the designer, that ultimately becomes a reality^9^. Not only does this introduce some difficulty in terms of control, we may also need to direct our attention to the responsibilities of the designer in such cases^10^.

We suggest that to delve into voting systems is rather to skip ahead. While these are worthwhile ventures to engage in, further research may also be required to highlight and explain the means of identify sufficient grounding for these voting systems and principles. Furthermore, we may need to investigate how to imbue such an agent with the ability to correctly classify whether it has followed such a principle in a meaningful fashion^11^.

## 5. Conclusion

Borda voting shows promise as a potential solution to certain issues under conditions of normative uncertainty. The case of self-driving cars shows that, when given a choice, a moral parliament that adopts Borda voting as a rule seemingly behaves more in line with the desires of many individuals. The premise of Borda voting (namely, selecting an option that is preferable to many) has the advantage of seeking consensus among a larger group of individuals. However, the general approach of dealing with normative uncertainty through voting is not without its downsides. Its effectiveness depends on the capacity for formalization, which is notoriously difficult with certain types of ethics. There is also the problem of “garbage in, garbage out.” In short, while Borda voting may seem to be an effective way forward, we also need to take into account the potential problems that accompany the bigger picture, especially if we wish to exert effective societal control over such technologies.

## Data availability statement

Publicly available datasets were analyzed in this study. This data can be found at: https://goo.gl/JXRrBP.

## Author contributions

KA performed the analysis and wrote most of the code. SK wrote the manuscript. All authors contributed to manuscript revision, read, and approved the submitted version.

## Footnotes

^1^Normative uncertainty is perhaps more aptly equated to a discussion in theology of sin. There is a difference between formal sin and material sin—i.e., between knowingly doing wrong, and only doing wrong in the eyes of God—and many of the discussions of possible worlds and moral theories that coincide with normative uncertainty actually align quite well with this discussion (Sepielli, 2010).

^2^A repository containing all the code can be found here: https://gitlab.com/SietzeKuilman/normative-uncertainty-as-a-voting-problem.

^3^A simple analogy by which to understand individual credences in a moral parliament is the image of different drives. For example, the drives to save and protect lives and to behave lawfully may be contradictory in certain situations. The credences represent, in effect, how important these motivations are to the corresponding individual.

^4^The standard deviation observed in all cases was ~1. We direct the reader to our specific implementation for the exact numbers.

^5^We are well aware that ageism is also socially sensitive, but quality-adjusted life years are also somewhat acceptable considerations in the medical field. On this topic, we highly recommend reading (Lin, 2016) specifically on the notion of crash-optimization.

^6^The limits of form problem can be related to problems of context, such as the frame problem (Dennett, 1984), and to the ludic fallacy (Taleb, 2008).

^7^To explain, the lack of operationalization may be caused by a lack of knowledge or a lack of different alternatives given a situation. In the case of virtue ethics, we can, for example, ask whether there are virtuous agents on one side of the road or the other, as this is crucial in deciding which alternative to pick. Of course, obtaining access to information on which agents are virtuous is profoundly difficult.

^8^We would like to thank an anonymous reviewer for reminding us of this.

^9^We should note that designer intentions become extremely interesting if we introduce something like epistemic opacity.

^10^We should note that, aside from eccentric inferences, ensuring correct action given a credence may not be as easy as it seems, because certain ideas could be taken too literally. For example, a principle of not harming bystanders may result in allowing a car to drive into the ground floor of a crowded building in an attempt to a save a bystander. In such an example, the modeling of the necessary connections between the destruction of a building (as a result of a car striking it) and the death of bystanders is not a given.

^11^We would like to thank an anonymous reviewer for pushing us on this point.
